# TYPOLOGIES OF CAREGIVING NETWORKS AND OLDER ADULTS' QUALITY OF LIFE

**DOI:** 10.1093/geroni/igac059.1641

**Published:** 2022-12-20

**Authors:** Mengyao Hu, Vicki Freedman, Sarah Patterson

**Affiliations:** University of Michigan, Ann Arbor, Michigan, United States; University of Michigan, Ann Arbor, Michigan, United States; University of Michigan, Ann Arbor, Michigan, United States

## Abstract

This study identifies typologies of caregiving networks of older adults and their effects on older adults’ well-being and unmet needs in late life using the National Health and Aging Trends Study (NHATS). NHATS identifies the entire care network of older adults, cataloguing all activities each respondent assists with and the amount of time they spend helping, providing a unique opportunity to evaluate caregiving networks. Using network analysis and cluster analysis, we find that older adults have different types of caregiving networks including large and small networks with a primary caregiver and networks where caregivers share caregiving responsibilities more evenly. Size and structure of the networks are significantly associated with older adults’ well-being and unmet needs. These findings highlight the importance of considering the full care network in developing policies and programs to support family caregivers.

